# Semantic Fusion Algorithm of 2D LiDAR and Camera Based on Contour and Inverse Projection

**DOI:** 10.3390/s25082526

**Published:** 2025-04-17

**Authors:** Xingyu Yuan, Yu Liu, Tifan Xiong, Wei Zeng, Chao Wang

**Affiliations:** School of Mechanical Science and Engineering, Huazhong University of Science and Technology, 1037 Luoyu Road, Wuhan 430074, China; yuanxy@hust.edu.cn (X.Y.); yu_liu_d@hust.edu.cn (Y.L.); xiongtf@hust.edu.cn (T.X.); weizeng_m@hust.edu.cn (W.Z.)

**Keywords:** camera, 2D LiDAR, point-line feature, calibration, projection matrix, semantic segmentation

## Abstract

Common single-line 2D LiDAR sensors and cameras have become core components in the field of robotic perception due to their low cost, compact size, and practicality. However, during the data fusion process, the randomness and complexity of real industrial scenes pose challenges. Traditional calibration methods for LiDAR and cameras often rely on precise targets and can accumulate errors, leading to significant limitations. Additionally, the semantic fusion of LiDAR and camera data typically requires extensive projection calculations, complex clustering algorithms, or sophisticated data fusion techniques, resulting in low real-time performance when handling large volumes of data points in dynamic environments. To address these issues, this paper proposes a semantic fusion algorithm for LiDAR and camera data based on contour and inverse projection. The method has two remarkable features: (1) Combined with the ellipse extraction algorithm of the arc support line segment, a LiDAR and camera calibration algorithm based on various regular shapes of an environmental target is proposed, which improves the adaptability of the calibration algorithm to the environment. (2) This paper proposes a semantic segmentation algorithm based on the inverse projection of target contours. It is specifically designed to be versatile and applicable to both linear and arc features, significantly broadening the range of features that can be utilized in various tasks. This flexibility is a key advantage, as it allows the method to adapt to a wider variety of real-world scenarios where both types of features are commonly encountered. Compared with existing LiDAR point cloud semantic segmentation methods, this algorithm eliminates the need for complex clustering algorithms, data fusion techniques, and extensive laser point reprojection calculations. When handling a large number of laser points, the proposed method requires only one or two inverse projections of the contour to filter the range of laser points that intersect with specific targets. This approach enhances both the accuracy of point cloud searches and the speed of semantic processing. Finally, the validity of the semantic fusion algorithm is proven by field experiments.

## 1. Introduction

Mobile robots are widely used in various fields because of their small size and flexible operation [1,2,3,4]. Camera and LiDAR are the two most representative sensors in global localization and the simultaneous localization and mapping (SLAM) field of mobile robots [5,6,7,8]. Camera-based visual SLAM can make use of rich texture information in images [9], but it is easily interfered with by light. The measurement accuracy of distance is a little low, the algorithm is complex, and the calculation is large. The laser SLAM based on LiDAR can directly obtain the depth of the measuring point [10], but it is prone to degradation and failure in corridors, tunnels, and other scenes lacking morphological information. In large industrial workshops, the global positioning efficiency is low, and the positioning algorithm easily falls into local optimality. Because they are complementary in so many ways, their fusion can balance out each other’s major weaknesses. With 2D LiDAR and camera data association, the information perceived by the camera and the laser reflection point distance information collected by the LiDAR can be fused, meanwhile the LiDAR reflection point also has semantic information, thus improving the positioning accuracy and efficiency of the robot [11].

To achieve data fusion between LiDAR and cameras, it is essential to accurately estimate the correspondence of the data, specifically by calibrating the relative orientation and position of the two sensors. Consequently, the semantic information of objects captured by the camera can be accurately aligned with the LiDAR scanning points. Traditional LiDAR and camera calibration methods rely on targets with high accuracy (such as checkerboards) or targets with artificially designed special shapes [12], which have poor adaptability to the actual industrial environment, and usually require two steps, namely internal parameter calibration and external parameter calibration of the camera and LiDAR. Both calibration processes necessitate data collection and nonlinear minimization, which are time-intensive activities. Furthermore, the calculation and estimation errors generated in the first step will propagate to the second step, thereby impacting the accuracy of the final calibration results. Additionally, most existing calibration algorithms depend on line features in the environment and do not accommodate arc features. There are also methods to achieve data correspondence currently, such as using the distance information obtained by binocular vision [13,14] or RGB-D cameras to fuse with LiDAR [15]. However, if calibration is completed through these methods, the calculation of vision will be large, and it will also increase the cost of equipment.

After the calibration of the LiDAR camera, the existing methods of semantic segmentation of the LiDAR point cloud are mainly divided into two types: the projection matrix is used to project the laser point coordinates onto the image, and the corresponding laser point semantic information is conferred by falling in the objects’ bounding box obtained by the convolutional neural network [16]; or the camera and LiDAR scanning data can be clustered and then fused by feature-level fusion, decision-level fusion, or the neural network [17]. In the former approach, semantic information is derived by projecting LiDAR points into the image’s bounding box. A significant drawback of this method is its computational intensity, as it necessitates reprojecting a substantial number of laser points into the image to determine which ones intersect with the target. Conversely, the latter approach relies on complex clustering and data fusion algorithms, facing significant limitations when handling large volumes of data and experiencing delays.

Based on the above analysis, this paper proposes a semantic fusion algorithm of LiDAR and cameras based on contour and inverse projection, including an adaptive LiDAR and camera calibration method and a fast and accurate semantic segmentation algorithm of laser point clouds. Aiming at the problem that traditional LiDAR and camera calibration methods rely on targets, have accumulated errors, and large calculation, a target-less calibration method based on environmental targets is designed, including linear and elliptic (circular) environmental targets. In order to improve the accuracy of image feature extraction, Fast R-CNN [18], a deep learning image extraction algorithm, is combined with the traditional image feature extraction algorithm to obtain a more accurate target contour feature equation. To solve the problems that the existing LiDAR point cloud semantic segmentation methods have a lot of reprojection calculation, limited accuracy, and require complex clustering and data fusion algorithms, a LiDAR point cloud semantic segmentation algorithm based on contour inverse projection is proposed, which greatly reduces the computation amount and improves the accuracy of LiDAR point cloud semantic segmentation.

The main contributions of this paper are as follows:

(1) In contrast to [19,20], whose calibration method relies on special targets and line features, a target-less calibration method based on random environmental targets is proposed in this paper. Two sets of point-line geometric constraints can be derived from a single photo. Circular or elliptical targets are also utilized in the calibration process. The calibration matrix is computed using multiple pairs of point-line constraints, reducing the number of targets required and enhancing adaptability to complex industrial environments. Additionally, the algorithm directly establishes data correspondence between 2D LiDAR and camera images, reducing cumulative error and computational complexity compared to the two-step calibration method.
(2) Different from [21,22], a unique inverse projection curve is obtained by projecting the contour of the target on the image back to the laser scanning plane. Based on the properties of the inverse projection curve, a semantic segmentation algorithm based on the target inverse projection curve is further proposed. The method is specifically designed to be versatile and applicable to both linear features and arc features, which significantly broadens the range of features that can be utilized in various tasks. This flexibility is a key advantage, as it allows the method to adapt to a wider variety of real-world scenarios, where both types of features are commonly encountered. By leveraging this adaptability, our method enhances the performance of calibration and semantic segmentation tasks, enabling more robust and accurate results across different environments and settings. The ability to seamlessly integrate linear and arc features improves the overall applicability of the approach, making it more generalizable and effective for practical use. Compared to existing semantic segmentation methods for LiDAR point clouds, the proposed algorithm requires only one or two projections to filter the laser points that correspond to a specific target, which reduces computational load while enhancing the accuracy of point cloud searches and the speed of establishing semantic targets.
(3) The effectiveness of the proposed semantic fusion algorithm of LiDAR and cameras based on contour and inverse projection is verified by experiments.

## 2. Related Works

### 2.1. Calibration of LiDAR and Camera Data

The calibration methods for LiDAR and cameras are generally categorized into target-based and target-less methods, which aim to extract the corresponding parameters of both sensors. By observing features or objects in the overlapping field of view, feature correspondence between the two sensors is established using geometric constraints, or the motion information from both sensors is utilized to maximize the interaction information and determine the calibration parameters. Target-based calibration methods usually require a high-precision calibration object, such as a specific polygon board or checkerboard designed manually. These types of calibration boards are relatively easy to detect for both sensors. At the same time, it is necessary to move the calibration plate to multiple positions and transform multiple angles to extract the feature correspondence between 2D LiDAR and the camera, so as to establish geometric constraints to solve the relative transformation relationship between them. The geometric constraints commonly used in the calibration process are mainly point-plane constraint, point-line constraint, point-point constraint, and line-plane constraint. Zhang et al. realized the external parameter calibration method of a 2D LiDAR and monocular camera by observing the checkerboard from multiple angles [12]. Firstly, the relative location between the camera and the target is estimated, and the laser points that fall on the chessboard find their corresponding coordinates in the camera coordinate system, and the vector is constructed. The point-plane geometric constraint is formed with the normal vector of the chessboard plane to obtain the linear solution of the calibration parameters. The nonlinear optimization is then performed by minimizing the reprojecting error of the laser points and taking the linear solution as the initial value. After that, target-based calibration studies such as checkerboard calibration boards were mainly focused on loss function optimization [23,24] and the introduction of new geometric constraints [25]. Due to the high accuracy requirements of the fabrication of reference targets such as calibration boards, which cannot be obtained anytime and anywhere, this kind of method is not convenient for on-site calibration or online calibration. At the same time, the internal parameter calibration of the camera, the external parameter calibration of the camera, and the LiDAR in the final calibration will result with cumulative errors.

In the field of LiDAR and camera calibration systems, research often involves the integration of heterogeneous sensor data to achieve precise environmental perception and navigation. These integrations necessitate the use of specific terminology to accurately convey the methodologies involved. In this paper, we use the term “target-less” to describe a calibration approach that does not rely on predefined physical targets [26,27]. In the context of LiDAR and camera calibration, “target-less” refers to techniques that leverage natural environmental features or ambient data for calibration. This method is especially advantageous in dynamic or uncontrolled environments where setting up and maintaining calibration targets is impractical. For example, in autonomous driving applications, a “target-less” calibration method allows the vehicle to continuously and adaptively calibrate its LiDAR and camera systems using natural features such as buildings, road markings, and other environmental objects. This ensures that the sensor systems remain accurately calibrated in real time without the need for manual recalibration. By using the term “target-less”, we clearly convey the innovative nature of this approach, distinguishing it from traditional target-based calibration methods.

The target-less calibration methods of LiDAR and cameras do not need to design accurate targets manually. They usually use some regular features or objects in the environment to achieve calibration, which reduces the design requirements for targets. Indeed, sometimes new sensors are added to the robot platform in order to extend the robot’s functionality, or existing sensors are disassembled and assembled again after maintenance or replacement. In this case, people should be able to effectively obtain the calibration parameters of the LiDAR and camera without the need for professional knowledge. Letting workers in the workshop or assembly line calibrate the sensors of the robot by themselves through environmental targets will greatly reduce their workload and calibration time. The target-less calibration method is more convenient and fast for calibration. Yang et al. used an infrared filter to directly recover the LiDAR scanning point cloud in the camera image so that an accurate correspondence between the two sensors could be established [28]. At the same time, they used V-shaped objects in the environment, such as points on the intersection line of two walls, to generate point-to-point geometric constraints. By this correspondence, they cast the calibration problem as a perspective n-point problem (PNP) for a calibrated camera or a planar homography problem for a camera that has not been calibrated. Briales and Gonzalez-Jimenez proposed a LiDAR and camera calibration method based on point-to-point geometric constraints by directly using intersection points in the scene [29]. They estimate the pose of the two sensors with respect to the corner of the scene separately, and then integrate the information from the two sensors to solve the calibration parameters. Zhang et al. used the column feature extraction of indoor structures, derived the projection matrix equation between LiDAR and camera according to the position relationship of points on the line, and optimized the calibration results after eliminating the points with large reprojection errors [30]. The existing calibration methods based on environmental features rely on infrared filter or line features to find corresponding points and can do nothing for the arc features captured by the camera. In this regard, the purpose of this paper is to reduce the restrictions on environmental features in the target-less calibration method. It can also use regular arc features (such as spheres, circle targets, etc.) to complete the calibration and improve the adaptability of the target-less calibration method.

### 2.2. Semantic Segmentation Algorithm for Laser Point Cloud

The cognitive map was proposed in 2007 [31], and it is believed that images contain semantic information, which can help robot localization in point cloud maps. With the development of computer technology and robot-intelligent technology, a large number of deep learning algorithms combined with SLAM products sprung up. For the method of building semantic maps, the general semantic target extraction for images needs to use deep learning algorithms such as R-CNN, YOLO [32], or SSD [33] to identify and extract the target in the image, and then combine the obtained information to give semantic information to the point cloud on the obtained point cloud map of reaction distance to construct semantic targets. Semantic information segmentation methods for point clouds are usually divided into two categories: one is the method only through vision, and the other is the method through the fusion of vision and LiDAR data. The existing mainstream methods of constructing semantic targets through only vision use monocular vision [34] and take multiple photos for depth estimation by moving the camera or setting up cameras at different positions to observe the target image in different positions in the camera image plane. The method of binocular vision is similar to monocular vision, which is composed of two cameras. Through the principle of parallax, the three-dimensional object is seen, and the length, width, and depth information of the object are obtained.

After fusing LiDAR and camera data, the convenience and high accuracy of LiDAR distance acquisition significantly reduce the computational complexity of constructing point cloud maps. Currently, the projection matrix is typically employed to map all laser point coordinates onto the image [35,36], and the corresponding laser point semantic information is given by falling into the bounding box of the target detection image obtained by the convolutional neural network. Alternatively, the laser point information of the detected object can be obtained using visual recognition and clustering algorithms for 2D LiDAR scanning data [37]. Additionally, the location of the target object can be predicted using the Kalman filter, as well as decision-level and feature-level fusion algorithms [38]. The former assigns semantic information by projecting the LiDAR points into the bounding box of the image target recognition map. In this method, a substantial number of laser points must be reprojected onto the image to identify the range of points that hit the target, and the LiDAR point cloud must undergo filtering processes, such as clustering. At the same time, limited by the target recognition algorithm based on deep learning, the target box is larger than the actual target size, and the laser points that hit the target within the bounding box can be captured, which makes the semantic information of the laser point cloud imprecise. The latter requires complex clustering and data fusion algorithms, and in the face of a large amount of data, the real-time performance is reduced, and the limitations are large. To this end, for industrial mobile robots, point cloud semantic segmentation and point cloud matching need to be performed directly, quickly, and at scale. How to reduce the calculation amount of LiDAR point cloud semantic endowment, while at the same time abandoning clustering and data fusion in the face of a large number of laser points to maintain real-time performance and quickly and accurately find the laser point cloud that hits the target is the research direction of this paper.

## 3. Algorithm Framework and Notation

**Figure 1:**
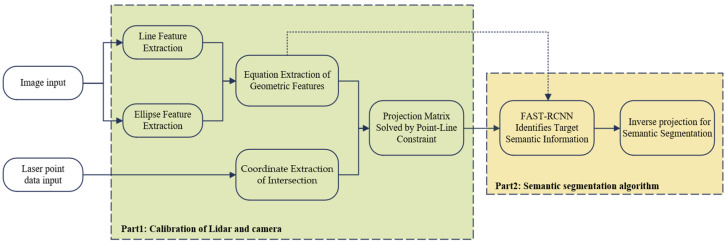
Semantic fusion algorithm of 2D LiDAR and camera based on contour and inverse projection.

In order to improve the adaptability and operation of the LiDAR and camera calibration methods, and to reduce the amount of calculation given by the laser point cloud semantics and improve its real-time performance, the algorithm as shown in Figure 1 is proposed.

Meanwhile, some notations and basic formulas used in this paper are defined. The position of a point in the corresponding coordinate system is denoted by superscript, for example, a point P under the LiDAR coordinate system is $P^{L}$, however P in the camera coordinate system is $P^{C}$. Additionally, $P^{W}={\left[X^{W},Y^{W},Z^{W}\right]}^{T}$ is the coordinate in the world coordinate system, and similarly, $P^{C}={\left[X^{C},Y^{C},Z^{C}\right]}^{T}$ is the coordinate of point P in the camera coordinate system.

**Figure 2:**
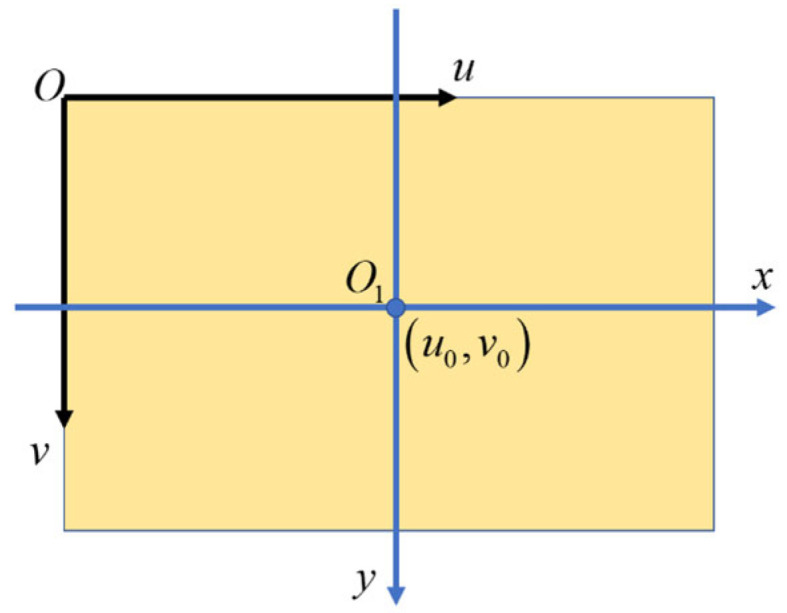
The coordinates of points on the image.

Since the image plane of the camera is a 2D plane, as shown in Figure 2, the coordinates of points on the image can be expressed as p=[u,v], which is the image coordinates in pixel units with the origin O of the vertex on the upper-left corner of the image, and the physical coordinates of pixels can be expressed as p=[x,y]. The pixel coordinate of the optical center $O_{1}$ of the camera is $\left[u_{0},v_{0}\right]$. If the physical dimensions of the pixel are dx and dy, respectively, the following equation is given:

(1) $$\left[\begin{array}{l} u\\ v\\ 1 \end{array}\right]=\left[\begin{array}{lll} \frac{1}{dx} & 0 & u_{0}\\ 0 & \frac{1}{dy} & v_{0}\\ 0 & 0 & 1 \end{array}\right]\left[\begin{array}{l} x\\ y\\ 1 \end{array}\right]$$

In images, we mainly use a coordinate system with units of pixels. A 2D line can be represented by $L={\left[a,\;b,\;c\right]}^{T}$, where a, b, c are the parameters of the line equation au+bv+c=0, respectively. For a conic curve in an image, we can define a matrix A to represent it as follows:

$$A=\left[\begin{array}{lll} a_{1} & a_{2} & a_{3}\\ a_{2} & a_{4} & a_{5}\\ a_{3} & a_{5} & a_{6} \end{array}\right]$$

where $a_{1},a_{2}\ldots a_{6}$ are the parameters in the conic equation:

$$a_{1}u^{2}+2a_{2}uv+2a_{3}u+a_{4}v^{2}+2a_{5}v+a_{6}=0.$$

## 4. Calibration of LiDAR and Camera Data

### 4.1. Image Features Extraction

**Figure 3:**
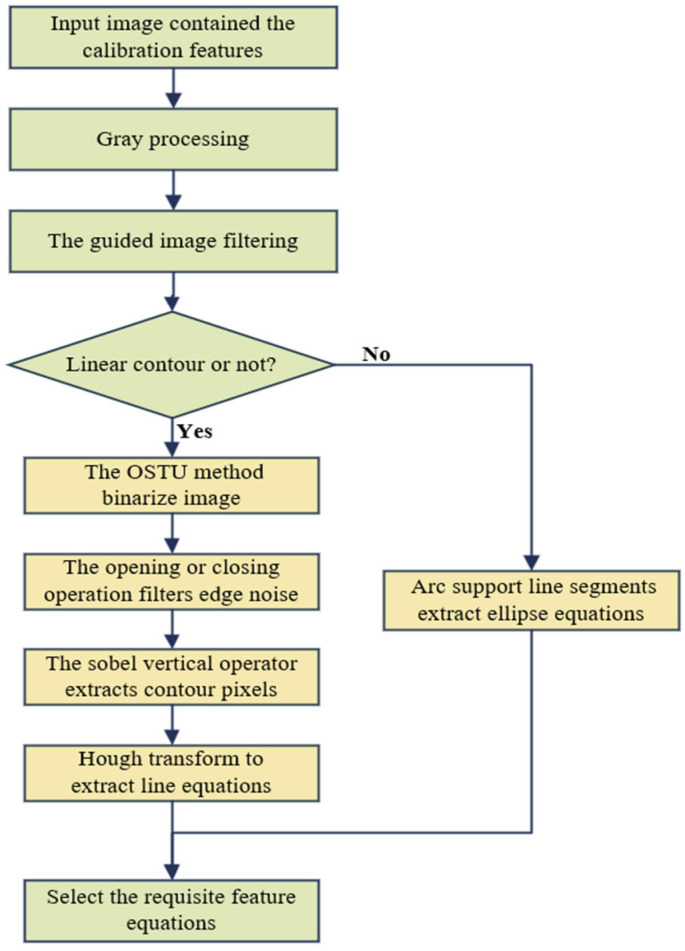
Image feature extraction algorithm.

From the framework of the LiDAR and camera semantic fusion algorithm in Section 3, it is clear that accurately obtaining the contour of the target in the image and its corresponding equation is essential to the calibration process. However, accurately extracting the contour is challenging due to various environmental noises and inherent limitations in camera design. To mitigate the impact of environmental and electronic noise on feature extraction, we developed the image processing workflow, as illustrated in Figure 3.

First, we convert the image to grayscale. Given the significant noise present in the external environment and during the shooting process, we use a guided image filter to smooth the image while preserving edge integrity. The guided filter is a neighborhood operation widely used for edge-preserving filtering. When the guided image matches the input image, their edges remain identical. Compared to Gaussian-filtered images, the guided filter better preserves edge integrity.

The methods for extracting contour pixels and geometric equations vary based on the type of target. For targets defined by straight-line contours, binarizing the image and extracting the target area is essential for quickly locating the target position. As the targets in this paper are environmental road signs with dark foregrounds against their surroundings, we utilize the Otsu method [39] to automatically set the threshold for extracting the foreground image, enhancing target area accuracy. The Otsu algorithm effectively controls thresholding by calculating the gray histogram properties of the entire image to automatically select a global threshold for image binarization.

The image after binarization will produce some noise on the edge of the target, which will make the edge extraction and line feature extraction inaccurate. Moreover, the target is in the dark foreground area, and the value of most pixels will become 0 after binarization. Therefore, we adopt the closing operation and select appropriate structuring elements to dilation and then erosion to make the boundary of the target purer and reduce the interference of noise.

Finally, for targets with linear characteristics, since the laser scanning plane of 2D LiDAR is a horizontal plane, the line intersecting with the laser scanning plane is generally perpendicular to the horizontal plane or tilted at a certain angle. When the target is within the range that can be observed by both LiDAR and the camera, the edge line of the target in the image meets this condition. Therefore, for the edge lines of the image that are valuable for calibration, we use the Sobel vertical operator for edge detection to reduce the horizontal distribution of pixels, so as to reduce the calculation of line detection and improve the speed of calibration.

According to Section 3, we need to obtain the geometric parameters of the edge lines of the targets on the image in the pixel coordinate system. Because the edge extraction by the Sobel operator only gets the sparse pixels distributed on the edge, it still needs to further filter the pixels and fit the line. Hough transform is one of the most suitable methods for line detection in images. If there are multiple pixels in the image, the curve transformed into Hough space intersects at one point, which means that the multiple pixels satisfy a line equation. Through this principle, the line feature in the image can be searched, and the line equation in the pixel coordinate system can be given. At the same time, Hough transform transforms the line equation into the form of polar coordinates (ρ,θ). If a point (u,v) on the image falls on the line, it must satisfy: ρ=ucosθ+vsinθ. Multiple lines may be extracted from the actual image, but only the vertical line edge is needed in the calibration process of this paper, so the distance threshold can be set in multiple candidate lines and the required target line equation can be manually selected by clicking.

For elliptical objects (such as circular road signs and spheres), they are elliptical in the image plane. After image denoising, it can be seen from Section 3 that the elliptic equation also needs to be extracted. At present, the stability, robustness, efficiency, and accuracy of the ellipse detection algorithm are not high in the case of edge clutter, motion blur, illumination, occlusion, and noise in the image. While the calibration method implemented in this paper requires frequent ellipse detection, the requirements for positioning accuracy and speed put higher limits on the ellipse detection algorithm. At present, the main ellipse detection algorithms are mainly divided into two types: ellipse detection based on Hough transform and edge following. For ellipse detection, the two-dimensional space formed by two parameters of a straight line is changed into a five-dimensional space formed by five parameters of an ellipse (center coordinates, major axis, minor axis, and deflection angle) in Hough space. In the statistics of common points, because of the five-dimensional space, the calculation is very large and the memory consumption is also large, so it is not practical to apply it to practice directly. Although there have been many studies to improve and optimize the ellipse Hough transform, the effect is still not satisfactory. On the other hand, compared to Hough transform, edge following improves the speed of ellipse detection, but it sacrifices positioning accuracy and robustness. Recently, researchers found that the LSD algorithm [40] can be viewed as a versatile detector that can detect line segments and ellipses. The detection of arc-supported line segments is improved with the original LSD algorithm [41], which is a fast and high-quality extraction algorithm, and it can directly extract the elliptic equation from the denoised grayscale image.

The ellipse extraction algorithm based on arc-supported line segments follows a four-step ellipse detection framework: formation of arc-supported groups, generation of initial ellipse sets, clustering, and candidate verification. Through these four steps, the positioning accuracy and efficiency of the extraction of elliptic equations can be guaranteed, and the final result is more robust. The refinement of the self-calibration helps to improve accuracy. Similar to line extraction, ellipse extraction encounters the challenge of detecting an excessive number of ellipses, necessitating the filtering of the target ellipses. By using the target’s position in the image and the semi-major axis size as preliminary selection criteria, we can derive the desired ellipse equation by regulating the number of supporting points on the ellipse contour and considering the importance of the spanning angle.

### 4.2. Coordinate Extraction of Intersection Between Laser Scanning Plane and Target Contour

We assume that there is a square or circular target in the real world that can be detected by LiDAR and there is an intersection line l between that and the laser scanning plane. This intersection line intersects the edge of the target at points P and Q, respectively. When the LiDAR scans, there must be a series of point cloud distributions at the intersection line l. We let the first beam hitting the calibration plate be I, and the last be II.

**Figure 4:**
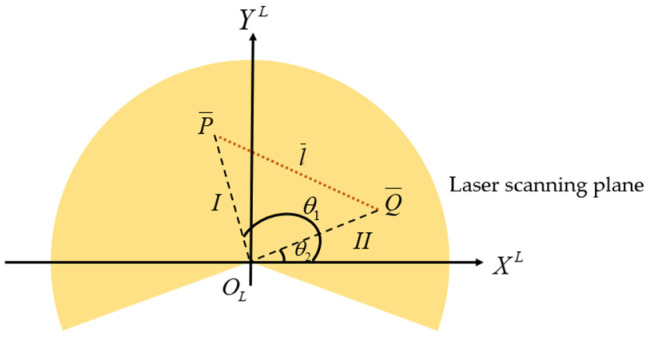
Schematic diagram of laser scanning.

Due to the limitations of LiDAR design principle, the collected data are sparse and have a certain amount of noise, and the laser points formed near the intersection line cannot form a straight line. In order to obtain the line equation of the intersection line in the LiDAR coordinate system, the fitted line $\bar{l}$ is obtained by fitting the laser points near the intersecting line l through the least square method. The angle values $\theta_{1}$ and $\theta_{2}$ returned by I and II are used to construct line $O_{L}\bar{P}$ and line $O_{L}\bar{Q}$ to cross-fit line $\bar{l}$ to point $\bar{P}$ and point $\bar{Q}$, respectively, as shown in Figure 4. Through this method, the coordinate values of the two points $\bar{P}$, $\bar{Q}$, $\left[X_{\bar{P}}^{L},Y_{\bar{P}}^{L}\right]$, and $\left[X_{\bar{Q}}^{L},Y_{\bar{Q}}^{L}\right]$ can be calculated.

The above method of extracting points on the edge is ideal, because it does not consider that the angle of the laser emitted by the LiDAR is discrete, and laser I and laser II cannot accurately fall on the edge of the target. LiDAR has a certain angular resolution α such that it can only detect the points θ=kα(k=1,2,3,⋯). If we use a LiDAR angular resolution of 0.48°, with a measurement range of 2 m, the interval between each two laser beams is about 9.84 mm. This indicates that, assuming that our LiDAR is highly accurate, the noise is negligible, and the camera and image processing algorithm can perfectly detect the edge, the point $\bar{P}$ may be 0~9.84 mm away from the actual edge point P. Therefore, in order to reduce this error, we design the following method.

If the polar coordinate of the actual edge point P in the LiDAR coordinate system is [θ,d], because there is an angular resolution α in LiDAR, the LiDAR may not accurately detect the point P, but it may detect one of its closest point P, whose polar coordinate is [kα,d’]. We define the angle error as ε, with $\varepsilon =\left|k\alpha -\theta \right|$.

In this paper, a method is adopted to improve the accuracy of data processing. The average error can be reduced to α/4, and the range of the error is limited to [0,α/2]. The implementation of this method is relatively convenient. For the nearest point $\bar{P}=[k\alpha ,d’]$ of any target edge, we can change it to [(k+0.5)α,d’’’] or [(k−0.5)α,d’’’] according to the direction of the detected edge. We can identify the range of the edge point by observing the sudden change in the distance value in the LiDAR point cloud. If the adjacent edge point $\bar{P}$ and point [(k+1)α,d’’] both hit the target, it means that the angle of the actual edge point must be within the range (k−1)α≤θ≤kα, so we modify the polar coordinates of the nearest boundary point to [(k−0.5)α,d’’’]. Similarly, if both $\bar{P}$ and [(k−1)α,d’’] hit the target, the coordinates change to [(k+0.5)α,d’’’]. By simply adjusting the angle of the nearest point of the laser edge to move α/2 to the actual angle, the angle error falls within the range of [−α/2,α/2], the absolute value of the error falls within the range of [0,α/2], the expectation of the error is α/4, and the angle error is reduced. In the case of larger angular resolution or more boundary point extraction times, the angle error is reduced. The improvement of the accuracy of the extracted points is more obvious.

### 4.3. Projection Matrix Construction and Solution

Camera imaging involves mapping real-world objects onto a 2D imaging plane, the point P in the real world is located in the world coordinate system, and the coordinate represents its position in the real world. Since the choice of the world coordinate system is related to the use scene, in order to facilitate the data fusion between the camera and the LiDAR, we overlap the world coordinate system and the LiDAR coordinate system. In order to project the point P onto the camera image plane coordinate system, the first step is to convert the point from the world coordinate system to the camera coordinate system. The X direction of the camera coordinate system is parallel to the image column direction (i.e., u direction), the Y direction is parallel to the image row direction (i.e., v direction), and the Z direction is the direction of the lens optical axis pointing out.

The second step is to project the 3D coordinates $P^{C}$ in the camera coordinate system onto the image plane. This process can be represented by the following equation:

(2) $$Z^{C}\left[\begin{array}{l} x\\ y\\ 1 \end{array}\right]=\left[\begin{array}{llll} f & 0 & 0 & 0\\ 0 & f & 0 & 0\\ 0 & 0 & 1 & 0 \end{array}\right]{\left[X^{C},Y^{C},Z^{C},1\right]}^{T}$$

Without considering image distortion, Equations (1) and (2) are combined together, which can be obtained as follows:

(3) $$\begin{array}{l} Z^{C}\left[\begin{array}{l} u\\ v\\ 1 \end{array}\right]=\left[\begin{array}{lll} \frac{1}{dx} & 0 & u_{0}\\ 0 & \frac{1}{dy} & v_{0}\\ 0 & 0 & 1 \end{array}\right]\left[\begin{array}{llll} f & 0 & 0 & 0\\ 0 & f & 0 & 0\\ 0 & 0 & 1 & 0 \end{array}\right]\left[\begin{array}{ll} R_{L}^{C} & t_{L}^{C}\\ 0 & \;1 \end{array}\right]P^{L}=KT_{L}^{C}P^{L}\\ K=\left[\begin{array}{lll} \frac{1}{dx} & 0 & u_{0}\\ 0 & \frac{1}{dy} & v_{0}\\ 0 & 0 & 1 \end{array}\right]\left[\begin{array}{llll} f & 0 & 0 & 0\\ 0 & f & 0 & 0\\ 0 & 0 & 1 & 0 \end{array}\right],T_{L}^{C}=\left[\begin{array}{ll} R_{L}^{C} & t_{L}^{C}\\ 0 & 1 \end{array}\right] \end{array}$$

where f is the focal length of the camera, $R_{L}^{C}$ and $t_{L}^{C}$ are the 3×3 orthogonal rotation matrix from the LiDAR coordinate system to the camera coordinate system and the 3×1 translation vector, respectively, K is the camera internal parameters matrix, and $T_{L}^{C}$ is the extrinsic parameters matrix of LiDAR and the camera.

Points in the LiDAR coordinate system need to be transformed to camera coordinate representation by a series of transformations, including rotation, translation, scaling, etc., and then project to pixel coordinates by the camera intrinsic matrix. However, the laser points of 2D LiDAR are only distributed on the laser scanning plane, and their $Z^{L}$ coordinates are identical to 0, so the transformation process can be concisely regarded as a 2D projective transformation between two planes.

When the point P falls on the LiDAR scanning plane, reasonably, we can define the laser scanning plane as the $Z^{L}=0$ plane in the LiDAR coordinate system. Therefore, the coordinate of point P is ${\left[X^{L},Y^{L},0\right]}^{T}$. If we merge the internal parameter matrix and external parameter matrix of the camera, let $\sigma =KT_{L}^{C}$ and use $\sigma_{i}$ to represent the i column of σ, then the P coordinate is put into Equation (3):

(4) $$Z^{C}\left[\begin{array}{l} u\\ v\\ 1 \end{array}\right]=\sigma \left[\begin{array}{l} X^{L}\\ Y^{L}\\ 0\\ 1 \end{array}\right]=\left[\sigma_{1}\;\sigma_{2}\;\sigma_{3}\;\sigma_{4}\right]\left[\begin{array}{l} X^{L}\\ Y^{L}\\ 0\\ 1 \end{array}\right]=\left[\sigma_{1}\;\sigma_{2}\;\sigma_{4}\right]\left[\begin{array}{l} X^{L}\\ Y^{L}\\ 1 \end{array}\right]=H\left[\begin{array}{l} X^{L}\\ Y^{L}\\ 1 \end{array}\right]$$

At this time, the coordinates of the point P in the image and its coordinates in the LiDAR coordinate system are $p={\left[u,v,1\right]}^{T}$ and $P^{L}={\left[X^{L},Y^{L},1\right]}^{T}$, respectively. Meanwhile, point P falls on the laser scanning plane and its $Z^{L}$ coordinate is always 0, p and $P^{L}$ can be regarded as homogeneous coordinates in the corresponding coordinate system, respectively, and Equation (5) can be rewritten as follows:

(5) $$p=\frac{1}{Z^{C}}HP^{L}$$

Equation (5) shows that the point coordinates in the 2D LiDAR coordinate system can be projected to the image plane by a homography matrix H.

From the above, the one-to-one correspondence is established between the coordinates of the points on the image and those in the LiDAR coordinate system. The homography matrix H has eight degrees of freedom and can be estimated by four sets of corresponding points [42].

Since general industrial cameras are used in this paper, and the laser points of 2D LiDAR are not visible in the images of general cameras, it is difficult to implement the point-point geometric constraints. Therefore, the feature-based target-less calibration method is adopted in this paper. The point-line geometric constraint is utilized to construct the corresponding relationship between point (which is the intersection of the LiDAR scanning plane and the contour of the target) and the equation of the contour in the image so as to obtain the calibration parameters of the LiDAR and the camera.

**Figure 5:**
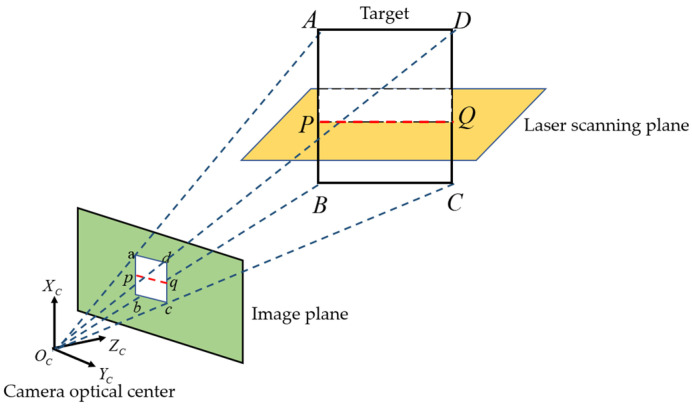
Schematic diagram of the target imaging on the camera image plane.

As can be seen from Section 4.1, when the target is placed in the overlapping field of view of the camera and the LiDAR, the target ABCD will form the corresponding image abcd. At the same time, there will be an intersection line l between the laser scanning plane and the target plane. This intersection line will intersect with the boundary of the target at two points P and Q, and these two points have the corresponding images p,q in the image plane, as shown in Figure 5.

For the point p on the image, it must be on the line ab, and we use $\chi_{ab}$ to denote the line ab, then

(6) $$\chi_{ab}^{T}p=0.$$

Using Equation (6), we can deduce that

(7) $$\frac{1}{Z^{C}}\chi_{ab}^{T}HP=0.$$

Similarly, for the circular target, the laser scanning plane still has an intersection line l and two intersection points P and Q with the target plane, and the contour of the circular target shown on the image is an ellipse A. As defined in Section 3,

(8) $$p^{T}Ap=0.$$

Using Equation (6), we obtain the following:

(9) $$P^{T}H^{T}AHP=0.$$

When Equations (8) and (10) know the coordinates of point P in the LiDAR coordinate system, only the homography matrix H is unknown. A set of point-line correspondences can provide a constraint. Since the homography matrix H has eight degrees of freedom, at least eight sets of corresponding geometric constraints are required to solve it.

Suppose we collect N sets of data, and since each set of data has two sets of point-line geometric constraints, we collected 2N sets of point-line correspondences. For the i-th group of point-line constraints, if the coordinate of the intersection point $P_{i}$ detected in the LiDAR coordinate system is $\left[X_{i}^{L},Y_{i}^{L}\right]$, the corresponding boundary $\chi_{i}$ or $A_{i}$ can be captured on the image. For the line boundary $\chi_{i}=[a_{i},b_{i},c_{i}]$, the point coordinates are put into Equation (8) as follows:

(10) $$[a_{i},b_{i},c_{i}]\left[\begin{array}{l} h_{11},h_{12},h_{13}\\ h_{21},h_{22},h_{23}\\ h_{31},h_{32},h_{33} \end{array}\right]\left[\begin{array}{l} X_{i}^{L}\\ Y_{i}^{L}\\ 1 \end{array}\right]=0$$

where $h_{ij}$ denotes the i-th row and the j-th column of H. Equation (10) is broken down as follows:

(11) $$S_{i}h=0$$

where

$$\begin{array}{l} S_{i}=[a_{i}X_{i}^{L},a_{i}Y_{i}^{L},a_{i},b_{i}X_{i}^{L},b_{i}Y_{i}^{L},b_{i},c_{i}X_{i}^{L},c_{i}Y_{i}^{L},c_{i}]\\ h={\left[h_{11},h_{12},h_{13},h_{21},h_{22},h_{23},h_{31},h_{32},h_{33}\right]}^{T} \end{array}.$$

When 2N groups of point-line correspondences are provided, 2N groups of equalities (11) can establish a homogeneous matrix with respect to h, as follows:

(12) Sh=0

where S is the 2N×9 matrix.

In fact, there may be many factors in the experimental process, so the invalid solution in Equation (13) will not have any exact solution except H=0. Therefore, we search for the optimal solution with the minimum value of the following equation:

(13) $$\begin{array}{l} h=\underset{h}{\arg\min}{\left\|Sh\right\|}_{2}\\ s.\;t.{\left\|h\right\|}_{2}=1 \end{array}.$$

There is a closed-form solution to this problem [42], which can be found using singular value decomposition (SVD).

In the case of the circular target, the coordinates of the point $P_{i}$ are taken into Equation (9) as follows:

(14) $$\left[X_{i}^{L},Y_{i}^{L},1\right]H^{T}AH\left[\begin{array}{l} X_{i}^{L}\\ Y_{i}^{L}\\ 1 \end{array}\right]=0.$$

Because this equation is nonlinear, in order to calculate the matrix H, we can use the closed-form solution obtained by the line boundary target as the initial value and use the Levenberg–Marquardt algorithm to optimize the following equation nonlinearly:

(15) $$H=\underset{H}{\arg\min}\sum_{i=1}^{2N}{\left(\left[X_{i}^{L},Y_{i}^{L},1\right]H^{T}AH\left[\begin{array}{l} X_{i}^{L}\\ Y_{i}^{L}\\ 1 \end{array}\right]\right)}^{2}.$$

Since the difference of the v coordinates on the image of the two points where the LiDAR hits the edge of the target will usually be within a certain range λ, we can add constraints:

$$s.\;t.\frac{[h_{21},h_{22},h_{23}][X_{2i}^{L},Y_{2i}^{L},1]’}{[h_{31},h_{32},h_{33}][X_{2i}^{L},Y_{2i}^{L},1]’}-\frac{[h_{21},h_{22},h_{23}][X_{2i-1}^{L},Y_{2i-1}^{L},1]’}{[h_{31},h_{32},h_{33}][X_{2i-1}^{L},Y_{2i-1}^{L},1]’}\leq \lambda$$

where points $P_{2i}$ and $P_{2i-1}$ are two points that hit the same target contour. The nonlinear solution of the homography matrix H can then be obtained.

At this point, the solution of the LiDAR and camera projection matrix (that is, the calibration parameters) has been completed. We can define the distance between the pixel position of the fitted intersection on the image and the corresponding line as the reprojection error. Due to the existence of various noises and processing errors, some points may have large errors and affect the experimental results. Therefore, we will eliminate the outliers to optimize the projection matrix. If the reprojection error of a data point is more than twice the mean, we will exclude it and recalculate H to obtain the optimized projection matrix.

## 5. Semantic Segmentation Algorithm for Laser Point Cloud

### 5.1. Projective Geometry and Inverse Projection of Contours

According to the calibration process of LiDAR and the camera in Section 4, there is a transformation relationship of Equation (5) between laser points and camera pixel points, which shows that there is a homography matrix between them and satisfies a one-to-one correspondence. When the relative position of LiDAR and the camera is fixed, the targets within the overlapping field of view of LiDAR and the camera are observed, and the correspondence between them can be regarded as a projective mapping from the LiDAR scanning plane to the image plane (that is, the LiDAR scanning plane can be regarded as the top view, and the picture taken by camera can be regarded as the main view).

First, we define three kinds of association relations: points on a line, points on a plane, and lines on a plane. When we regard the plane π as the set of all points and lines associated with it, we denote it as $\pi =\left[P,l\right]$, where P is the set of points and l is the set of lines. Conversely, we can also view a point P as the set of all planes and lines associated with it, denoted $P=\left[\pi ,l\right]$. We refer to the set defined in this way as the set of P.

For a set whose center is point O, any point P on plane π determines a line in the set of O when O is not on the plane π. For any line l’ on the plane π, it is determined that one of the planes $\pi^{\prime }$ is in the set of O. We denote the newly obtained set $O^{\prime }=\left[\pi^{\prime },l^{\prime }\right]$ as the projection of the plane π on the set of O.

For the same point O that is not incidental with the plane π, the plane $\pi_{0}$ passing through O will intersect with π in a line $l_{O\pi }$, and the line l passing through O intersects the plane at a point $P_{Ol}$, then we denote the newly obtained set $\pi *=\left[l_{O\pi },P_{Ol}\right]$ as the intercept of π on the set of O.

However, in the ordinary Euclidean plane, when the line and plane pass through points O and parallel to the π at the same time, they cannot find the associated points, lines, or planes on the plane to form a projective or intercept relationship. In order to make the above situation conform to the central projection rule, we introduce the infinite origin and the infinity line into the Euclidean plane as the extended Euclidean plane so that two parallel lines must intersect at the infinite origin and the planes of the two planes must intersect at the infinity line. In this way, the projection and the intercept satisfy all cases, making the central projection bijective.

**Figure 6:**
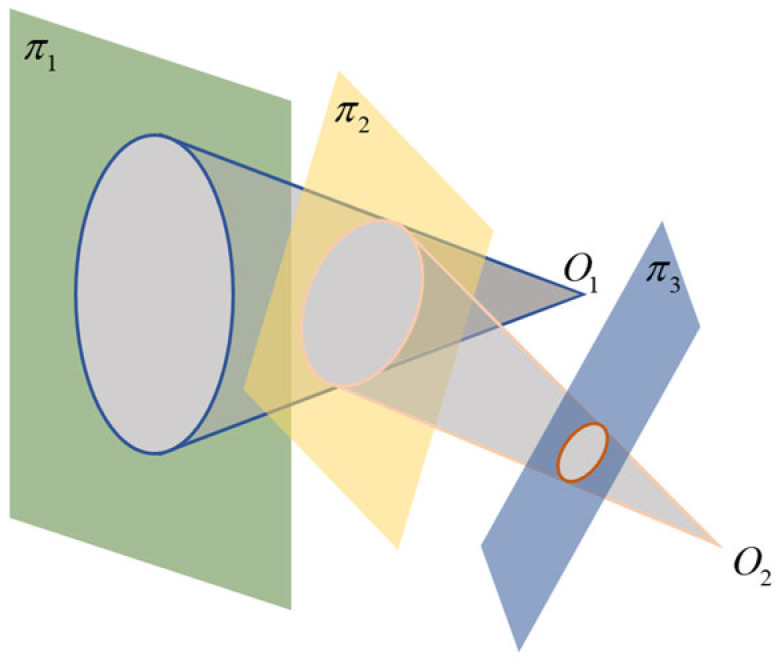
The schematic representation of the projective mapping relationship.

A projective map is a map through a finite number of central projections such that the projective plane π becomes the projective plane $\pi^{\prime }$. It can keep the collinearity of projective space (plane, line) and the intersection ratio of four collinear points constant. As shown in Figure 6, we project the circular curve on the plane $\pi_{1}$ through the center of the point light source $O_{1}$. Through the projective map, $f:\pi_{1}\to \pi_{2}$, we can obtain the ellipse on the plane $\pi_{2}$. The ellipse on the plane $\pi_{3}$ is then obtained by projecting it through the center of $O_{2}$. In this process, a projective mapping relationship is determined, namely, $f:\pi_{1}\to \pi_{3}$. According to the properties of projective mapping, when there is a correspondence relationship between four points, only the transformation matrix can be obtained to satisfy the projective mapping relationship, and the projective mapping of the two faces is invertible.

**Figure 7:**
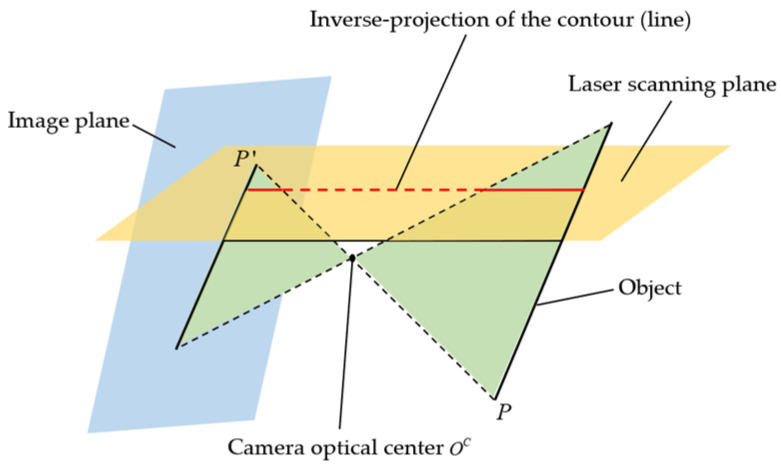
The projective relationship of line between the Lidar scanning plane and the camera image plane.

At this time, we consider the imaging relationship between the LiDAR scanning plane and the camera image plane, as shown in Figure 7. For the linear target, in the case of the limited distance of the camera to the light source, the target appears as a line on the image plane. Considering the principle of projective geometry as described above, the green area in Figure 7 is the projection of the target plane to the set of the camera optical center, and the image of the target on the image plane is obtained by combining the camera image plane with the set of the camera optical center. If expressed in mathematical language, the target can be seen as a large number of points, take one point P, and the line $O^{C}P$ is a line in the set of $O^{C}$ determined by the point P on the target plane; this constitutes a mapping, $f_{1}:P\to O^{C}P$; and the point P is the preimage of the line $O^{C}P$ under the projection. P’ is a point in the camera image plane determined by truncating a line $O^{C}P$ in the set of $O^{C}$; this constitutes another mapping, $f_{2}:O^{C}P\to P^{\prime }$; and point P’ is the image of line $O^{C}P$ under the intercept. Then, the process of central projection can be expressed as follows:

(16) $$f_{2}(f_{1}(P))=(f_{2}{}^{\circ}f_{1})(P)=P^{\prime }.$$

If the central projection process is carried out on all the points constituting the target, the image of the target on the image plane can be obtained. In Section 4, we deduce the homography matrix by using the geometric relationship of more than eight pairs of point-line constraints between the laser scanning plane and image plane. The projections that exist on these two planes can be viewed as the perceptivities of the camera center. H can be regarded as a projection transformation matrix [42]. Due to the dual relationship between points and lines in the projective map, the line L=[a,b,c] on the image can be mapped to the laser scanning plane as L′ by the following equation:

L’=LH.

The actual mapping is shown in Figure 7. For conic curves, the above mapping can be rewritten as follows:

$$A’=H^{T}AH.$$

**Figure 8:**
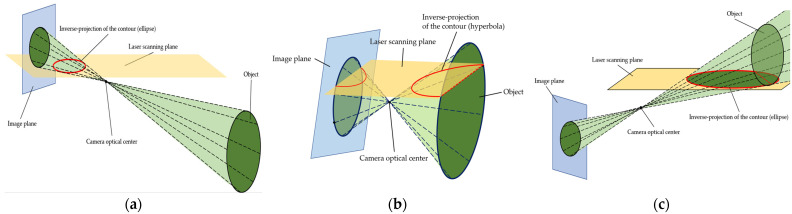
Projection relationships between three situations of laser scanning planes and camera image planes. (**a**) The inverse projection is a line. (**b**) The inverse projection is a hyperbola. (**c**) The inverse projection is a ellipse.

The target plane forms a central projection on the laser scanning plane through the family of camera optical center $O^{C}$. As the object transitions from below to above the camera’s centerline, the inverse projected curve transitions from an ellipse to a hyperbola and back to an ellipse, while the conic curve’s center, derived from the circular object’s contour via inverse projection, travels from the point at infinity to the camera’s optical center and back to infinity. As can be seen from Figure 8a, the contour of the object on the image plane is projected as an ellipse through the center of the camera, and the center projection on the laser scanning plane is an ellipse. While the circular object moves, the center projection on the laser scanning plane becomes a hyperbola, as shown in Figure 8b. It is also possible to form an ellipse on the laser scanning plane when the object moves above the horizontal line of the camera’s optical center, as shown in Figure 8c.

With the above mapping relationship, we can capture the edge of the target by the image feature extraction algorithm, and inverse-project the edge lines to the laser scanning plane by the inverse of the projective transformation matrix so as to assign semantic information to the relevant LiDAR points.

### 5.2. Improved Image Feature Extraction

Using the principles described in Section 5.1, we can reverse project the contours of the objects in the image onto the laser scanning plane. This paper has stable calibration results for LiDAR and the camera, and how to perform data fusion between them is also an important work. Assigning the semantic information in the picture to the laser points in the LiDAR coordinate system is an important part of building the semantic map.

Established methods exist for obtaining semantic information from image pixels. Typically, deep learning algorithms such as R-CNN, YOLO, or SSD are employed to extract object features within images, establishing frames and providing semantic information for the pixels inside. However, image sensors have limited accuracy in distance perception. Meanwhile, LiDAR laser points can only provide distance information and cannot identify the objects the laser interacts with. The fusion of these two complementary sensors will accelerate point cloud matching for mobile robots in large scenes and enhance global positioning accuracy and robustness.

In this paper, the Fast R-CNN image recognition algorithm is used. After Fast R-CNN completes image recognition, the captured target and the corresponding bounding box are displayed on the image. Since the environment of semantic target construction for mobile robots is usually a real environment, such as a workshop, compared with the environment of LiDAR and camera calibration, noise and interference will be significantly increased. Additionally, since the ellipse extraction algorithm based on arc-support line segment is built on gray images, if the gray difference between the target and the surrounding environment is not large, it will affect the actual geometric equation extraction results. At this point, if we perform image feature extraction on the whole image as described in Section 4.1, many unwanted geometric features and equations may be obtained on the image. At the same time, it will be extremely difficult to screen the geometric features that need to be extracted, and the accuracy of the extracted geometric equations will also be reduced. In order to avoid this situation, we can use the bounding box obtained after image recognition for image segmentation. Usually, the marked bounding box identified by Fast R-CNN cannot fully fit the object boundary, which is wider than the contour of the target, and the frame is generally square, not to say it cannot fit the circular object boundary. By listing the pixel set in the bounding box separately and performing image feature extraction as described in Section 4.1 within the listed pixel set, environmental noise and interference can be effectively isolated and accurate contour geometric equation extraction can be achieved. Then, the geometric equation is restored to the original image according to the coordinates of the bounding box, and the contour equation of the target is obtained.

### 5.3. Semantic Segmentation Algorithm Based on Contour Inverse Projection

After obtaining the contour curve equation of the target, we inversely project the boundary line to the laser scanning plane according to the principle described in Section 5.1. If the target is a square object, inversely projecting its two vertical boundaries will result in two intersecting lines in the LiDAR coordinate system. If the target is a circular object, because the object cannot be completely perpendicular to the optical axis in reality, it will appear as an ellipse on the image plane, and its inverse projection to the LiDAR coordinate system will be a conic curve. Considering the complexity of the conic curve equation, the calculation amount of the point cloud will be greatly affected. Usually, the optical center of the camera and the LiDAR are arranged on a vertical line as far as possible. In the case of conic curve mapping, the distance of the target setting is generally much larger than the horizontal distance between the optical center of the camera and the LiDAR, and far away from the center of the hyperbola. For just screening laser points, the angle error of the tangent and asymptote of the hyperbola can be ignored. Therefore, the asymptote of the hyperbola is used to replace the hyperbola as the inverse projection of the target boundary and the tangent through the origin is used instead of the conic curve as the inverse projection of the target boundary. When the hyperbola in the LiDAR coordinate system is represented in Section 3, let the asymptote of the hyperbola be y=kx+b, and put it into the hyperbola equation as follows:

(17) $$(a_{1}+2a_{2}k+a_{4}k^{2})x^{2}+(2a_{2}b+2a_{3}+2a_{4}kb+2a_{5}k)x+a_{4}b^{2}+2a_{5}b+a_{6}=0.$$

According to the properties of hyperbola,

(18) $$\left\{\begin{array}{l} a_{1}+2a_{2}k+a_{4}k^{2}=0\\ 2a_{2}b+2a_{3}+2a_{4}kb+2a_{5}k=0 \end{array}\right..$$

According to the above equations, the two asymptote equations of the hyperbola can be obtained. In the case that the mapping is an ellipse, since most of the laser points hitting the target fall within the ellipse, we can use the ellipse tangent passing through the origin instead of the inverse projection of the target boundary to limit the angle range. When the ellipse in the LiDAR coordinate system is represented in Section 3, let the tangent of the ellipse be y=kx, and put it into the elliptic equation as follows:

(19) $$\left(a_{1}+2a_{2}k+a_{4}k^{2}\right)x^{2}+(2a_{3}+2a_{5}k)x+a_{6}=0.$$

Considering that it is a tangent line with only one intersection point with the ellipse, the discriminant Δ=0 can obtain the slope k of the tangent line and then obtain the elliptic tangent equation. At this time, both straight and circular targets can be represented by two intersecting lines in the LiDAR coordinate system.

In addition, when the target contour is irregular, the bounding box can also be used as the contour for inverse projection to determine the range of laser points hitting the target.

If the calibration parameters of the camera and the LiDAR are known, these two lines are obtained by the inverse projection of the object contour on the image. At the same time, n laser points hitting the target plane in the LiDAR coordinate system are $\left[\theta_{1},\theta_{n}\right]$, and the laser points hitting the object must be distributed in the intersection of the laser scanning plane and the target plane. Due to the properties of projective geometry, these laser points are also bound to fall between the inverse-projected lines in the LiDAR coordinate system. Therefore, we only need to search for the laser point whose scanning angle falls between the angles of the inverse projection lines, so as to determine the position of the laser point that hits the target and endow it with semantic information.

However, due to the problem of equipment placement and the structure of the LiDAR, the laser point hitting the target cannot completely reflect the target contour information (as described in Section 4.2). Moreover, the angle returned by the laser point is the counterclockwise angle when the origin is the vertex, and the *X*-axis is one side. While the inverse-projected line or asymptotic line is the angle converted by the slope. Due to the position deviation of the LiDAR center and the camera optical center, the intersection point of the two lines cannot coincide with the origin in practice. If only the angle constraint of a straight line or an asymptote is adopted, it cannot assign semantic information to all the target points that hit the target when the target is close to the LiDAR and camera.

In order to make the segmentation of semantic information more accurate, we need to establish a target point group for the laser points that hit the target which contains all the laser points that hit the target and has uniform semantic information. We first search for eligible laser points within the angle range of the two asymptotes. The angular resolution of the laser radar used in the experiment is 0.48°, and the Fast R-CNN image recognition algorithm is combined with the image feature extraction algorithm in the process of object boundary extraction, so the deviation caused by the inverse projection curve is small. Moreover, in practical applications, the optical center of the laser radar and the camera are usually set on a vertical line. Therefore, angle $\sigma_{1}$ and $\sigma_{2}$ can be used as the starting point to search for the angle of LiDAR points in a small range with the step size of the angle resolution α. From the observation of LiDAR point cloud data, it can be seen that the sudden change in LiDAR range reading generally occurs at the edge of the object, so we can set a certain threshold of sudden distance variation to detect the edge laser point by this method. If no laser point with a range mutation is detected in a small surrounding area with angle $\sigma_{1}$ and $\sigma_{2}$ as the starting point, it means that the corresponding feature in the image has not been hit by LiDAR. If we find a distance mutation between the first and last two points of the angle reading of the $\left[n_{1},n_{2}\right]$ frame of the LiDAR near the angle of the two lines, it is taken as the laser point on the edge of the object. The laser point range is divided through the above steps, and an array of 3×1 is generated at the same time. The first two numbers in the array represent the angle of the first and last laser point that generated the distance mutation, respectively. Between the two frame readings is the laser point that hit the target. The third column of the array represents the type label of the identified object, which is represented by characters and can be set freely. In this way, the corresponding semantic information is given to the laser point in the LiDAR coordinate system.

## 6. Experimental Verification and Analysis

In this part, we carry out actual experiments to verify the effectiveness of the proposed method.

### 6.1. Experimental Equipment and Environment

**Figure 9:**
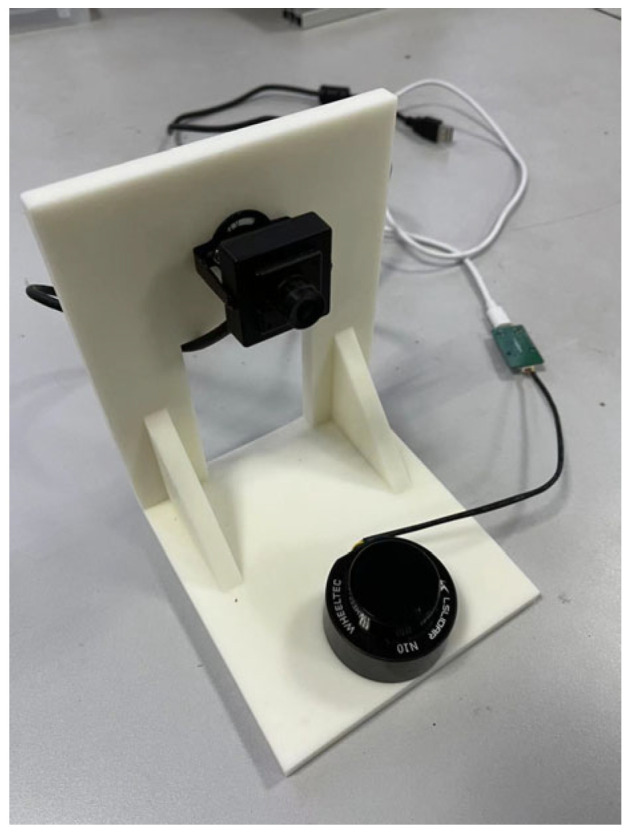
The relative position of the LiDAR and camera.

In order to verify the calibration algorithm of LiDAR and the camera and the semantic segmentation method proposed in this paper, the more common road targets are selected as experimental targets, and the experimental environment with debris and certain interference is selected to simulate the actual industrial environment, so as to reflect the accuracy of image processing. The camera used in this experiment is an ordinary industrial camera with a resolution of 720P and a pixel size of 3 μm×3 μm. The laser radar used is the single-line TOF laser radar of LSLIDAR, the scanning angle was 360°, the angular resolution was 0.48°, and the measurement accuracy was ±3 cm (0–6 m), ±4.5 cm (≥6 m). The relative positions of the camera and LiDAR are shown in Figure 9.

### 6.2. Experimental Results of Calibration and Analysis

**Figure 10:**
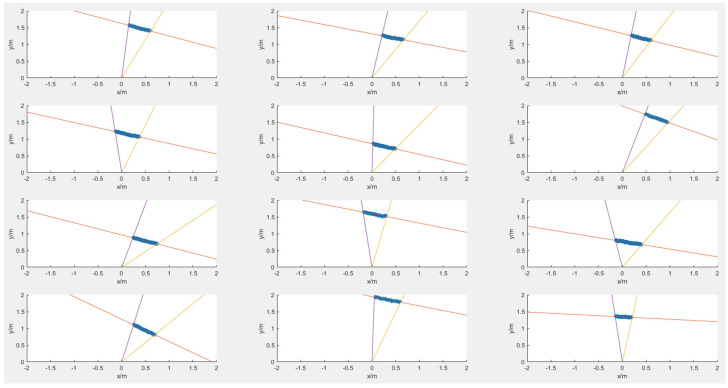
Point cloud of LiDAR hitting linear targets and fitting straight-lines. The purple line and the yellow line represent the lines between the target’s boundary point and the center of the lidar; the red line denotes the fitting line of the laser point cloud for the concentrated target.

Ensure that the points extracted from the images between the groups of data and the line equations between the groups of data are not linearly correlated, respectively, and the optical center of the camera is not in the extended targets plane. The tilt angle and position of the target are adjusted during the experiment. We took several groups of photos of linear targets, from which we selected 12 groups of photos of linear targets used for subsequent calculations. The point cloud fitting and intersection position of the LiDAR hitting the target are shown in Figure 10. Since there are generally two intersection points between the LiDAR scanning plane and the target plane, 24 groups of point-line constraints can be obtained from 12 groups of photos, and the projection matrix from LiDAR to camera can be obtained. The 24 groups of data collected for targets of line characteristics are shown in Table 1.

Based on the above data and the principle of solving the projection matrix, Equation (13) is listed, and the initial projection matrix H is obtained by singular value decomposition as follows:

$$H=\left[\begin{array}{ccc} -0.941491790438115 & -0.279218868357607 & 0.00386033374859088\\ -0.0704340778598327 & -0.148055012054731 & -0.0934517999080423\\ -0.000249985115967346 & -0.000763279769177976 & 9.85283234313479\;\times {\;10}^{-7} \end{array}\right].$$

**Figure 11:**
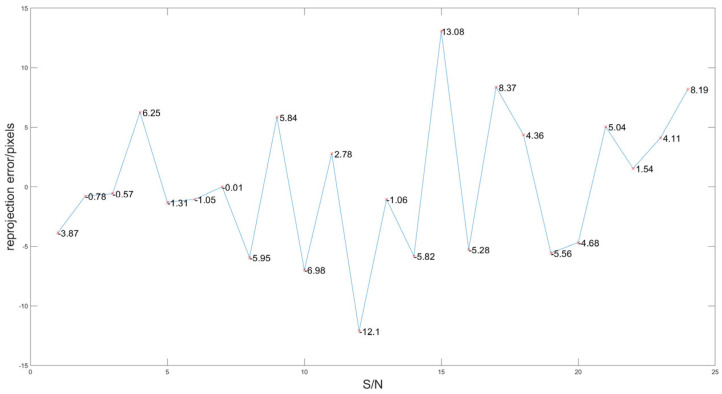
Reprojection error curve before optimization.

According to the definition of reprojection error in Section 4.3, we calculate the reprojection error of the above points through the initial projection matrix H as shown in Figure 11. The average of the absolute value of reprojection error is 4.7743 pixels.

By eliminating points where the reprojection error is more than twice the mean, the optimized projection matrix H is calculated as follows:

$$H=\left[\begin{array}{ccc} -0.941640057896292 & -0.282468741142702 & 0.0285118369522878\\ -0.0490771573053093 & -0.127151897621540 & -0.118893536187977\\ -0.000269735542930214 & -0.000753564019521682 & 2.81909082878981\times 10^{-5} \end{array}\right].$$

**Figure 12:**
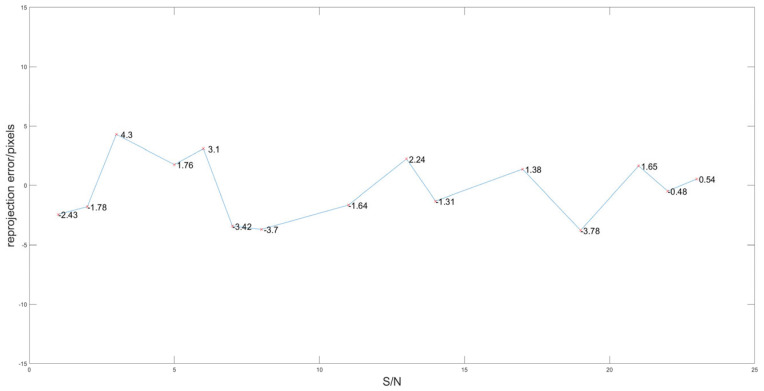
Reprojection error curve after optimization.

**Figure 13:**
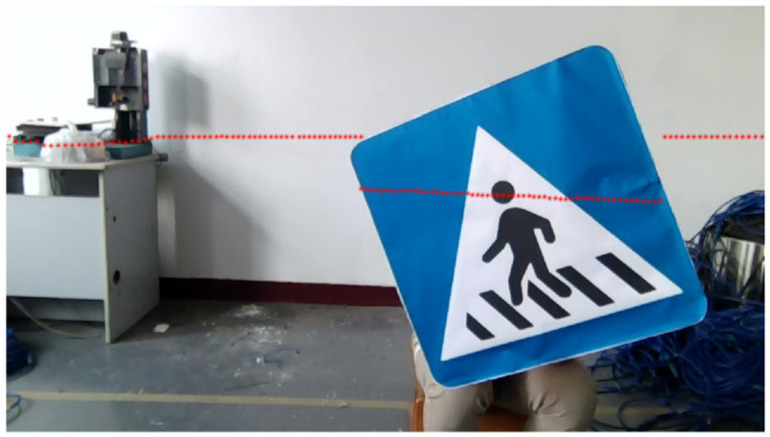
The result of projecting laser points onto the linear target image. The red points represent the positions of the laser points projected onto the image via the projection matrix.

Using the optimized projection matrix to reproject laser points, the average of the absolute value of reprojection error is 2.2333 pixels after optimization, which is 2.5410 pixels lower than that before optimization. The reprojection error is shown in Figure 12. The results shown in Figure 13 are obtained by projecting all laser points that hit the target onto the image.

**Figure 14:**
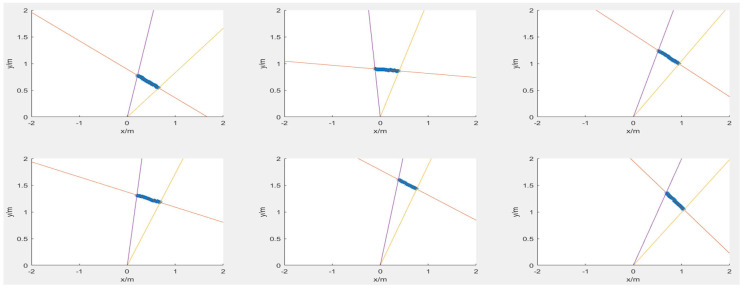
Point cloud of LiDAR hitting elliptic targets and fitting straight lines. The purple line and the yellow line represent the lines between the target’s boundary point and the center of the lidar; the red line denotes the fitting line of the laser point cloud for the concentrated target.

In order to reflect the adaptability of the calibration method, we change the relative position of the LiDAR and the camera slightly to simulate the equipment disturbance in the actual environment and use the circular object for calibration. The point cloud fitting and intersection position of the LiDAR hitting the target are shown in Figure 14, and the collected geometric features are shown in Table 2:

After obtaining the above data, we take the optimized projection matrix as the initial value and put it into Equation (15), when i=5, λ=6, and use the Levenberg–Marquardt algorithm to solve the problem. Align the first term of the obtained result with the initial value. The projection matrix is obtained as follows:

$$H=\left[\begin{array}{ccc} -0.941640057896292 & -0.0924436533552060 & -0.0662369018061970\\ -0.0699266866203810 & -0.113102699664438 & -0.123213620330822\\ -0.000282938355870838 & -0.000628813082252420 & -9.93786256821846\times 10^{-5} \end{array}\right].$$

After calculating the reprojection error, data with the reprojection error greater than twice the mean are excluded. After eliminating them, the projection matrix is recalculated as follows:

$$H=\left[\begin{array}{ccc} -0.941640057896292 & -0.0907027820699054 & -0.0678745965775377\\ -0.0649303621894133 & -0.104852095353131 & -0.131855795342972\\ -0.000274315305355119 & -0.000618303570427709 & -0.000110703470006810 \end{array}\right].$$

**Figure 15:**
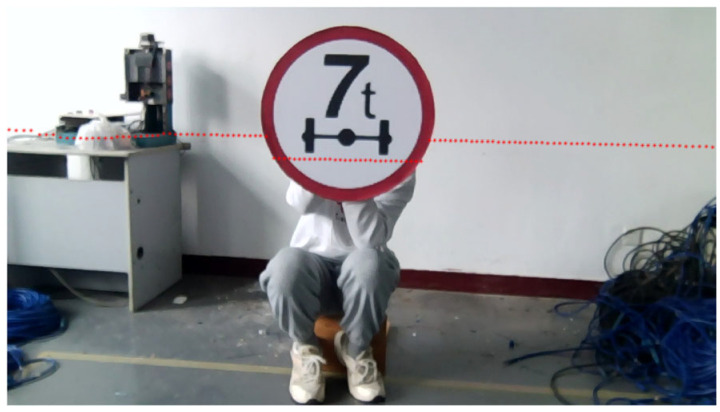
The result of projecting laser points onto the elliptic target image. The red points represent the positions of the laser points projected onto the image via the projection matrix.

The average reprojection error is 2.7837 pixels. The laser point reprojection effect is shown in Figure 15.

### 6.3. Comparison with Mainstream Algorithms

In order to verify the accuracy and robustness of our calibration algorithm, we compared the proposed algorithm with the target-based calibration method [25] and the calibration algorithm based on indoor environment characteristics [30] in the same scenario. The comparison of different calibration algorithms is shown in Table 3, the average reprojection error and error distribution interval are shown in Table 4, and the calibration times of different methods are shown in Table 5.

#### Explanation of Methods

Method [25]: This traditional approach uses predefined calibration targets placed in the environment to align LiDAR and camera data accurately. It offers high precision in controlled environments. However, it requires manual setup of targets and is prone to cumulative errors over time, especially in dynamic or varying conditions.

Method [30]: This “target-less” method leverages linear environmental features such as buildings or roads for calibration. It is particularly useful in feature regular settings where setting up targets is impractical. However, it may struggle in environments lacking distinct features, potentially leading to lower precision.

Our method: Our method utilizes line and arc-shaped features for the LiDAR calibration process. This approach enhances the use of environmental features and optimizing the precision of LiDAR sampling point extraction. Furthermore, the calibration matrix is optimized non-linearly using arc-shaped features, resulting in improved calibration accuracy. Despite its lower effectiveness in high-speed dynamic areas, this method provides significant adaptability and precision across various environments.

As illustrated in the table, our proposed method demonstrates a lower average reprojection error and superior accuracy compared to the other two methods. Additionally, our method exhibits a narrower error distribution interval, enhanced robustness in complex environments, and improved calibration performance. In contrast to Method [25], our approach eliminates the calibration of the camera’s internal parameters and directly establishes the relationship between camera pixels and LiDAR points, minimizing cumulative errors associated with both internal and external parameter calibration. Compared to Method [30], we also achieve a significant improvement in accuracy. This improvement results from optimizing the method for extracting LiDAR point coordinates and applying a non-linear optimization using arc-shaped features, which not only reduces reprojection error, but also enhances the calibration algorithm’s adaptability to varying environmental conditions while expanding the range of geometric features used in the calibration process. However, in the introduction of additional nonlinear constraints, the final solution time will be slightly increased.

### 6.4. Experimental Results of Semantic Segmentation and Analysis

After realizing the calibration of the LiDAR and camera, the relative positions of the LiDAR and camera are kept constant and ready for semantic segmentation of the objects in the environment. There are objects such as balls and instruments in the experimental environment. Through different categories of objects in the experimental environment, the target laser point groups are set to different colors, as shown in Table 6.

**Figure 16:**
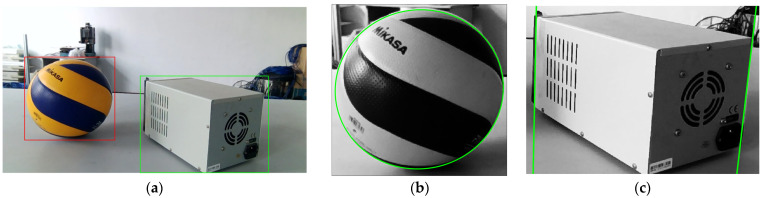
The results of image recognition and boundary extraction. (**a**) Results of image recognition: the red bounding box indicates the ball, while the green bounding box indicates the instrument. (**b**) Results of elliptic boundary extraction: the green ellipse represents the boundary of the ball. (**c**) Results of linear boundary extraction: the green lines represent the boundaries of the instrument.

Through object recognition and contour extraction in the field of view, set the threshold for contour extraction, and the camera can obtain the contour information in Figure 16.

**Figure 17:**
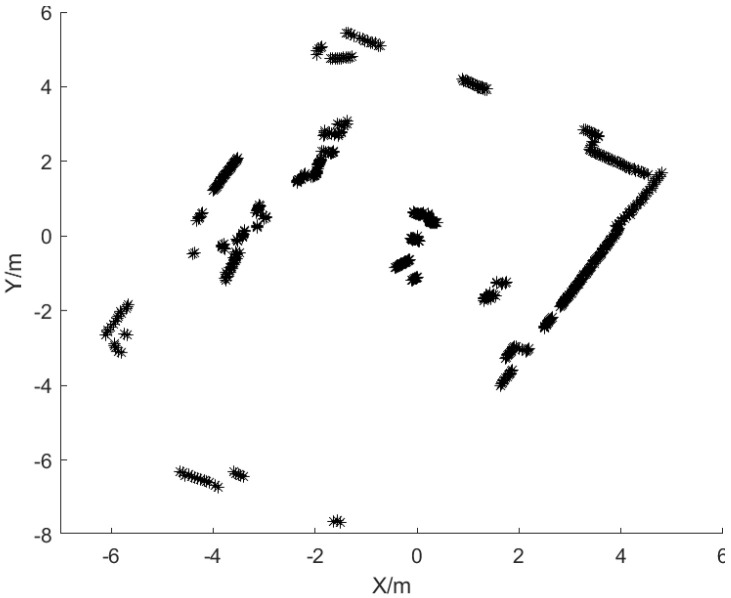
The laser point cloud of this experiment. The black asterisk represents the point cloud map generated by the LiDAR as it interacts with environmental targets.

The laser point cloud image obtained after LiDAR scanning the environment is shown in Figure 17.

**Figure 18:**
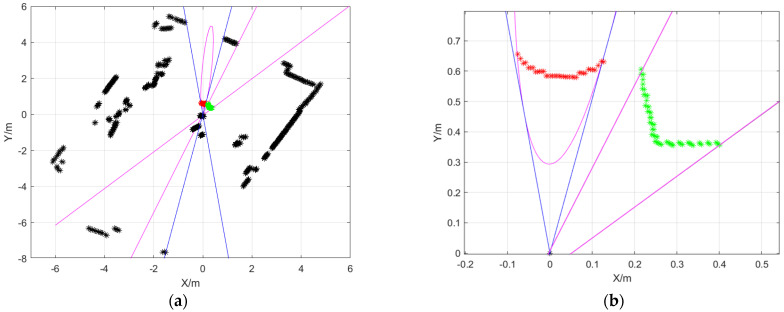
The results of semantic segmentation algorithm. (**a**) Original image: the magenta lines and ellipse indicate the outcome of the inverse projection of the target outline, while the blue lines represent the tangent lines of the ellipse at the origin. The black asterisk represents the point cloud map generated by the LiDAR as it interacts with environmental targets. The red asterisk denotes the laser point cloud that struck the ball, and the green asterisk denotes the laser point cloud that struck the instrument. (**b**) Enlarged view.

The projection matrix of LiDAR and the camera was used to inverse-project the object contour to the laser scanning plane, and the angle range of the two straight line boundaries was calculated as $\left[45.4909^{\circ },69.8830^{\circ }\right]$, and the angle range of the two ellipse tangent lines crossing the origin was $\left[78.8795^{\circ },97.4829^{\circ }\right]$. The angle segmentation was used through the algorithm in Section 5 to obtain that the laser beam hitting the instrument was [41,101,‘green’], and the laser beam hitting the sphere was [1,24,‘red’]∪[736,749,‘red’]. As shown in Figure 18, the magenta lines are the lines obtained by inverse-projecting the object contour to the laser scanning plane, and the blue lines are the tangent lines of the ellipse at the origin. The red point set is the target point cloud group obtained by the LiDAR hitting the sphere, and the green point set is the target point cloud group obtained by hitting the instrument.

If laser point reprojection is to be performed to endow semantic information to laser points falling in the bounding box, 750 times of reprojection calculations are needed, and the proposed method reduces the calculation to one or two calculations. At the same time, an object with circle or ellipse contour, such as a ball, cannot well conform to the target frame, and may cause misjudgment of the laser point that hits the target. The contour inverse projection adopted in this paper can avoid this problem.

The field experiments show that the calibration method of LiDAR and camera proposed in this paper can well meet the actual conditions, the reprojection error is small, and it has high flexibility. At the same time, the accurate position of the target identified in the environment can be found on the LiDAR point cloud, and the semantic information can be segmented. The calculation is small, the speed is fast, and the feasibility is high. When there are some sundry objects and interference in the experimental environment, the recognition of laser points that hit the target object can still maintain a certain accuracy.

### 6.5. Discussion and Future Planning

The proposed algorithm performs well in static or low-speed dynamic scenarios, but its effectiveness decreases in high-speed dynamic environments. This is primarily due to the temporal misalignment and motion blur affecting the accuracy of both image-based detection and point cloud association. To address this issue, specifically, we analyze the factors that contribute to the performance degradation and propose several potential strategies for improvement, including the following: 1. Motion compensation: introducing motion estimation techniques (e.g., optical flow or IMU integration) to compensate for rapid movement between frames. 2. Temporal fusion: utilizing sequential data and temporal filtering (e.g., Kalman filter or recurrent neural networks) to maintain detection continuity over time. 3. Higher frame rate sensors: Employing sensors with higher frame rates or lower latency to reduce the gap between object motion and data acquisition. We also highlight this limitation as an important direction for future research and plan to further explore robust multi-sensor fusion techniques to enhance performance under complex, high-speed conditions.

## 7. Conclusions

In this paper, a data calibration method for a camera and LiDAR based on environmental targets is proposed. This method can use the circle or ellipse feature for calibration, which improves the adaptability of the calibration method to different geometric features of the target object. At the same time, two sets of calibration data are obtained through one set of experiments, which reduces the number of experiments. The accuracy of the final calibration result is improved by improving the accuracy of the intersection coordinates of the laser scanning plane and the object contour. On this basis, this paper proposes a semantic segmentation method for LiDAR point clouds based on contour inverse projection. The method is specifically designed to be versatile and applicable to both linear features and circular features, which significantly broadens the range of features that can be utilized in various tasks. By leveraging this adaptability, our method enhances the performance of calibration and semantic segmentation tasks, enabling more robust and accurate results across different environments and settings. Compared with the existing methods, the proposed method only needs one or two projections to quickly determine the range of laser points that hit the target, which greatly reduces the calculation of laser point reprojection. Meanwhile, depending on the properties of the inverse projection curve, the laser point group that hits the target can be accurately found. Field experiments show that the reprojection errors of the LiDAR and camera calibration matrices obtained by using the line feature and the arc feature are all within 3 pixels, and the calibration results are consistent with the reality. The laser points of the target in the attack can be segmented accurately and given semantic information by using the contour inverse projection.

It is worth mentioning that while the proposed algorithm demonstrates excellent performance under typical environmental conditions, it faces challenges in scenarios where the target shapes cannot be recognized or in high-speed dynamic environments. These limitations highlight areas for future research, where we aim to explore and develop strategies to enhance the algorithm’s robustness and applicability in such complex situations.

## Figures and Tables

**Table 1:** Laser point coordinates and extraction line parameters of linear targets.

S/N	$X^{L}$	$Y^{L}$	a	b	c
1	0.6077	1.3984	0.9945	0.1045	−816
	0.1446	1.5715	0.9998	0.0175	−468
2	0.6678	1.1350	0.7314	−0.6820	−464
	0.2112	1.2595	0.7071	0.7071	−581
3	0.6021	1.1178	0.8988	−0.4384	−654
	0.1839	1.2626	0.4540	0.8910	−495
4	0.3736	1.0628	0.6947	−0.7193	−275
	−0.1396	1.2232	0.7431	0.6691	−374
5	0.4913	0.7056	1	0	−987
	0.0182	0.8574	0.9998	−0.0175	−385
6	0.9665	1.4957	0.9205	0.3907	−987
	0.4754	1.7439	0.9135	0.4067	−707
7	0.7442	0.6956	0.8829	0.4695	−1263
	0.2333	0.8803	0.9135	0.4067	−718
8	0.3107	1.5084	0.9994	−0.0349	−555
	−0.1861	1.6463	0.9986	−0.0523	−223
9	0.4115	0.6749	0.8910	−0.4540	−654
	−0.1470	0.8026	0.5000	0.8660	−371
10	0.6974	0.8088	0.9336	−0.3584	−924
	0.2475	1.1092	0.9397	−0.3420	−459
11	0.6062	1.7807	0.8480	0.5299	−733
	0.0517	1.9347	0.8572	0.5150	−468
12	0.2098	1.3290	0.4067	−0.9135	50
	−0.1505	1.3549	0.9063	0.4226	−324

**Table 2:** Laser point coordinates and extraction elliptic parameters of elliptic targets.

S/N	$X^{L}$	$Y^{L}$	A
1	0.6477	0.5456	$\left[\begin{array}{ccc} 95473.082 & 49.819 & -80878431.125\\ 49.819 & 100422.381 & -37546782.292\\ -80878431.125 & -37546782.292 & 72933959443.262 \end{array}\right]$
	0.2180	0.7751	
2	0.3871	0.8617	$\left[\begin{array}{ccc} 80073.262 & -947.628 & -30057494.007\\ -947.628 & 86654.733 & -27584299.603\\ -30057494.007 & -27584299.603 & 13354842773.671 \end{array}\right]$
	−0.1031	0.8991	
3	0.9402	0.9984	$\left[\begin{array}{ccc} 34549.867 & -429.213 & -29301322.186\\ -429.213 & 36219.536 & -10512356.668\\ -29301322.186 & -10512356.668 & 26865444783.932 \end{array}\right]$
	0.5272	1.2396	
4	0.6753	1.1803	$\left[\begin{array}{ccc} 35084.162 & -124.632 & -20399379.489\\ -124.632 & 35584.997 & -10162224.801\\ -20399379.489 & -10162224.801 & 13556248705.685 \end{array}\right]$
	0.2088	1.3124	
5	0.7494	1.4319	$\left[\begin{array}{ccc} 24656.896 & -208.467 & -15138285.074\\ -208.467 & 24767.309 & -4655803.187\\ -15138285.074 & -4655803.187 & \;9607674206.991 \end{array}\right]$
	0.3896	1.6011	
6	1.0446	1.0482	$\left[\begin{array}{ccc} 29799.785 & 532.522 & -27660662.803\\ 532.522 & 31344.116 & -8994848.139\\ -27660662.8027 & -8994848.139 & 27047385872.157 \end{array}\right]$
	0.6917	1.3522	

**Table 3:** Comparison of calibration algorithms.

Method	Description	Key Feature	Advantages	Disadvantages
Method [25]	Utilizes predefined calibration targets to align LiDAR and camera data (‘target-based’)	Checkerboard	High precision in controlled environments	Requires manual setup of targets, potential cumulative errors
Method [30]	Employs indoor structural features for calibration without predefined targets (“target-less”)	Line features	Adaptive to feature regular environments	Less precise in feature-sparse areas
Ours	Utilizes a variety of environmental morphological features for calibration and precision optimization (“target-less”)	Line features and arc features	High adaptability and precision in diverse environments; Improved feature utilization	Less effectiveness in high-speed dynamic areas

**Table 4:** Experimental results of reprojection error for different calibration methods.

	Method [25]	Method [30]	Ours
Average reprojection error/pixels	6.54	4.88	2.78
Error distribution interval/pixels	[4.13, 9.64]	[1.55, 6.20]	[1.49, 5.78]

**Table 5:** Calibration time of different calibration methods.

	Method [25]	Method [30]	Ours
Calibration time/s	0.82	0.85	1.03

**Table 6:** Objects in the environment and their corresponding semantic colors.

Object	Color
ball	red
instruments	green

## Data Availability

Data are contained within the article.
